# Healthy lifestyle change and all-cause and cancer mortality in the European Prospective Investigation into Cancer and Nutrition cohort

**DOI:** 10.1186/s12916-024-03362-7

**Published:** 2024-05-29

**Authors:** Komodo Matta, Vivian Viallon, Edoardo Botteri, Giulia Peveri, Christina Dahm, Anne Østergaard Nannsen, Anja Olsen, Anne Tjønneland, Alexis Elbaz, Fanny Artaud, Chloé Marques, Rudolf Kaaks, Verena Katzke, Matthias B. Schulze, Erand Llanaj, Giovanna Masala, Valeria Pala, Salvatore Panico, Rosario Tumino, Fulvio Ricceri, Jeroen W. G. Derksen, Therese Haugdahl Nøst, Torkjel M. Sandanger, Kristin Benjaminsen Borch, J. Ramón Quirós, Carlota Castro-Espin, Maria-José Sánchez, Amaia Aizpurua Atxega, Lluís Cirera, Marcela Guevara, Jonas Manjer, Sandar Tin Tin, Alicia Heath, Mathilde Touvier, Marcel Goldberg, Elisabete Weiderpass, Marc J. Gunter, Heinz Freisling, Elio Riboli, Pietro Ferrari

**Affiliations:** 1 International Agency for Research on Cancer (IARC-WHO), Lyon, France; 2 Cancer Registry of Norway (Kreftregisteret), Oslo, Norway; 3 Department of Clinical Sciences and Community Health, University of Milan, Milan, Italy; 4 Department of Medical Epidemiology and Biostatistics, Karolinska Institutet, Stockholm, Sweden; 5 Department of Public Health, Aarhus University, Aarhus, Denmark; 6 Danish Cancer Society Research Center, Copenhagen, Denmark; 7 Department of Public Health, University of Copenhagen, Copenhagen, Denmark; 8 Inserm, Université Paris Saclay, Institut Gustave Roussy, Team Exposome, Heredity, Cancer and Health, CESP UMR 1018, 94807 Villejuif, France; 9 Division of Cancer Epidemiology, German Cancer Research Center (DKFZ), Heidelberg, Germany; 10 Department of Molecular Epidemiology, German Institute of Human Nutrition Potsdam-Rehbruecke, Nuthetal, Germany; 11 Institute of Nutritional Science, University of Potsdam, Nuthetal, Germany; 12 German Center for Diabetes Research (DZD), Neuherberg, Germany; 13 Institute for Cancer Research, Prevention and Clinical Network (ISPRO), Florence, Italy; 14 Epidemiology and Prevention Unit, Fondazione IRCCS Istituto Nazionale dei Tumori di Milano, Milan, Italy; 15 Dipartimento di Medicina Clinica, Federico II University, Naples, Italy; 16 Hyblean Association for Epidemiological Research, AIRE ONLUS, Ragusa, Italy; 17 Centre for Biostatistics, Epidemiology, Department of Clinical and Biological Sciences, and Public Health (C-BEPH), University of Turin, Turin, Italy; 18 Julius Center for Health Sciences and Primary Care, University Medical Center Utrecht, Utrecht University, Utrecht, The Netherlands; 19 K.G. Jebsen Center for Genetic Epidemiology, Department of Public Health and Nursing, NTNU - Norwegian University of Science and Technology, Trondheim, Norway; 20 Department of Community Medicine, UiT The Arctic University of Norway, Tromsø, Norway; 21 Public Health Directorate, Asturias, Spain; 22 Unit of Nutrition and Cancer, Catalan Institute of Oncology-ICO, L’Hospitalet de Llobregat, Barcelona, Spain; 23 Nutrition and Cancer Group, Epidemiology, Public Health, Cancer Prevention and Palliative Care Program, Bellvitge Biomedical Research Institute-IDIBELL, L’Hospitalet de Llobregat, Barcelona, Spain; 24 Escuela Andaluza de Salud Pública (EASP), 18011 Granada, Spain; 25 Instituto de Investigación Biosanitaria ibs.GRANADA, 18012 Granada, Spain; 26 Centro de Investigación Biomédica en Red de Epidemiología y Salud Pública (CIBERESP), 28029 Madrid, Spain; 27 Department of Preventive Medicine and Public Health, University of Granada, 18071 Granada, Spain; 28 Sub Directorate for Public Health and Addictions of Gipuzkoa, Ministry of Health of the Basque Government, San Sebastian, Spain; 29 Epidemiology of Chronic and Communicable Diseases Group, Biodonostia Health Research Institute, San Sebastián, Spain; 30 Department of Epidemiology, Murcia Regional Health Council, IMIB—Arrixaca, Murcia, Spain; 31 Department of Health and Social Sciences, University of Murcia, Murcia, Spain; 32 Instituto de Salud Pública y Laboral de Navarra, 31003 Pamplona, Spain; 33 Navarra Institute for Health Research (IdiSNA), 31008 Pamplona, Spain; 34 Department of Surgery, Skåne University Hospital Malmö, Lund University, Malmö, Sweden; 35 Nuffield Department of Population Health (NDPH), University of Oxford, Oxford, England; 36 School of Public Health, Imperial College London, London, UK; 37 L’Institut national de la santé et de la recherche médicale (Inserm), Paris, France; 38 Université Paris Descartes, Paris, France

**Keywords:** Healthy lifestyle index, Composite score, Change score, Mortality, Cancer, Longitudinal, Prospective study

## Abstract

**Background:**

Healthy lifestyles are inversely associated with the risk of noncommunicable diseases, which are leading causes of death. However, few studies have used longitudinal data to assess the impact of changing lifestyle behaviours on all-cause and cancer mortality.

**Methods:**

Within the European Prospective Investigation into Cancer and Nutrition (EPIC) cohort, lifestyle profiles of 308,497 cancer-free adults (71% female) aged 35–70 years at recruitment across nine countries were assessed with baseline and follow-up questionnaires administered on average of 7 years apart. A healthy lifestyle index (HLI), assessed at two time points, combined information on smoking status, alcohol intake, body mass index, and physical activity, and ranged from 0 to 16 units. A change score was calculated as the difference between HLI at baseline and follow-up. Associations between HLI change and all-cause and cancer mortality were modelled with Cox regression, and the impact of changing HLI on accelerating mortality rate was estimated by rate advancement periods (RAP, in years).

**Results:**

After the follow-up questionnaire, participants were followed for an average of 9.9 years, with 21,696 deaths (8407 cancer deaths) documented. Compared to participants whose HLIs remained stable (within one unit), improving HLI by more than one unit was inversely associated with all-cause and cancer mortality (hazard ratio [HR]: 0.84; 95% confidence interval [CI]: 0.81, 0.88; and HR: 0.87; 95% CI: 0.82, 0.92; respectively), while worsening HLI by more than one unit was associated with an increase in mortality (all-cause mortality HR: 1.26; 95% CI: 1.20, 1.33; cancer mortality HR: 1.19; 95% CI: 1.09, 1.29). Participants who worsened HLI by more than one advanced their risk of death by 1.62 (1.44, 1.96) years, while participants who improved HLI by the same amount delayed their risk of death by 1.19 (0.65, 2.32) years, compared to those with stable HLI.

**Conclusions:**

Making healthier lifestyle changes during adulthood was inversely associated with all-cause and cancer mortality and delayed risk of death. Conversely, making unhealthier lifestyle changes was positively associated with mortality and an accelerated risk of death.

**Supplementary Information:**

The online version contains supplementary material available at 10.1186/s12916-024-03362-7.

## Background

The role of lifestyle in shaping our health outcomes is a critical area of study. Modifiable lifestyle factors such as tobacco and alcohol consumption, physical activity, diet, and body mass index (BMI) have been identified as important risk factors for non-communicable diseases including heart diseases, stroke, pulmonary diseases, and cancer [1]. These diseases are responsible for a considerable proportion of global mortality, accounting for 74% of the 55.4 million deaths recorded in 2019 [2]. Adherence to a healthy lifestyle is associated with extended life expectancy [3] and lower premature mortality [4].

Over the last years, investigations on individual lifestyle factors have been complemented by a more holistic approach, using a composite score, or a healthy lifestyle index (HLI) to combine lifestyle factors to measure adherence to a healthy lifestyle [5–7]. HLI has been linked to cancer incidence [8, 9], lymphoma [10], and the multimorbidity of cancer and cardiovascular disease [11] in the European Prospective Investigation into Cancer and Nutrition (EPIC) cohort. A review of 45 EPIC studies concluded that adherence to healthier lifestyle behaviours was consistently associated with lower cancer mortality [12]. Additionally, a systematic review and meta-analysis of 21 studies found that adherence to at least four healthy lifestyle behaviours combined was associated with 66% lower all-cause mortality, compared to unhealthier lifestyle profiles [13]. Another systematic review of 30 studies found that the healthiest lifestyle profiles were associated with 58% lower cancer mortality, compared to the least healthy [14].

However, nearly all studies on healthy lifestyle and mortality conducted thus far have relied on measurements obtained at a single point in time, usually at study recruitment. Inferences made from single-time-point measurements are based on hypothetical counterfactuals had healthier lifestyles been adopted in the first place, instead of observed changes in lifestyle behaviours within the same participant over time. Recently, we analysed the associations of HLI changes on risk of incident colorectal cancer [15] and lifestyle-related cancers [16]. Very few studies have investigated the impact of changing lifestyle behaviours on mortality using longitudinal exposure measurements and have only been completed on a national scale [17–19]. It is important to employ longitudinal data in the assessment of changing behaviours to inform public health recommendations and to decrease the burden of mortality due to modifiable risk factors.

In addition to modifiable lifestyle behaviours, social determinants of health, such as socioeconomic position (SEP), have been estimated to account for 30–55% of health outcomes [20]. SEP, combined with the other lifestyle factors, has been associated with premature mortality in several independent cohort studies. In a multi-cohort meta-analysis of 1.7 million participants, low SEP participants had greater mortality compared with those with high SEP, with the third highest population attributable fraction after smoking and physical inactivity [21]. Low SEP has also been associated with double the rate of all-cause mortality compared to high SEP in the UK Biobank and the US National Health and Nutrition Examination Survey (US NHANES) [22]. While the interplay of lifestyle with other social determinants of health, such as sex or smoking [23–25], has been repeatedly documented, the interaction between changing lifestyle behaviours and SEP and their impact on mortality remains largely unexplored.

Within the multi-national EPIC study, we used longitudinal exposure measurements on smoking status, alcohol intake, BMI, and physical activity, combined into an HLI, to estimate the association between changing lifestyle behaviours and all-cause and cancer mortality and to quantify the impact of lifestyle changes on mortality.

## Methods

### Study population and design

**Fig. 1:**
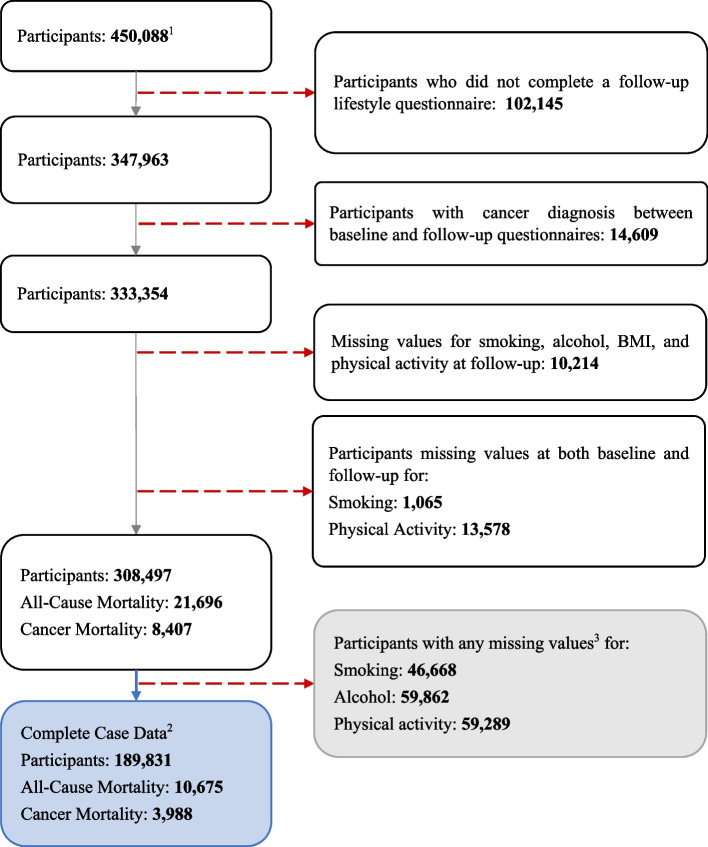
Flowchart of exclusion criteria for the study

EPIC is an ongoing prospective study, which enrolled 521,323 adults aged 35 to 70 from 23 centres in 10 European countries (Denmark, France, Germany, Greece, Italy, the Netherlands, Norway, Spain, Sweden, and the UK) between 1991 and 2000 [26]. Participants completed a baseline lifestyle questionnaire upon recruitment and a follow-up questionnaire, on average 7 years later. Of the 521,323 participants enrolled, we excluded participants from Greece for administrative reasons (*n* = 28,561), participants who did not complete a baseline questionnaire (*n* = 6342), participants with top or bottom 1% of the ratio of energy intake to energy requirement (*n* = 9607), participants who had no follow-up vital status (*n* = 1800), participants who ended follow-up before the second questionnaire due to death or censorship (*n* = 1378), participants with unknown date of death (*n* = 69), or participants with prevalent cancer cases at recruitment (*n* = 23,478). In addition, participants were excluded if they did not complete a follow-up lifestyle questionnaire (*n* = 102,125), if they received a cancer diagnosis in the time between completing the first and second questionnaire (*n* = 14,609), and if they were missing all follow-up lifestyle data (*n* = 10,214) or data for any one component of the HLI at both baseline and follow-up (*n* = 14,643), as detailed in Fig. 1. In summary, 308,497 participants were included in the analysis. All study participants provided informed consent to participate in the study, and ethical approval was obtained from the participating centres and ethics committees.

### Exposure assessment

The HLI score was calculated at baseline and follow-up as the sum of smoking history, alcohol intake, BMI, and physical activity. Each factor comprised a score ranging from 0 to 4, with low and high values corresponding to less and more healthy behaviours, respectively, and the total score ranging between 0 and 16 (Fig. S1). Changes in HLI were calculated as the difference in score between HLI at follow-up and HLI at baseline. HLI change was modelled as a continuous and categorical variable, i.e. improving (increasing HLI by more than one unit), stable (within a one-unit change), and worsening (decreasing HLI by more than one unit).

Data on diet was only available at baseline and was therefore not included in the HLI score and change analyses; however, baseline diet score was used as an adjustment factor in all analyses. The diet score was calculated based on the intakes of cereal fibre, red and processed meat, polyunsaturated to saturated fat ratio, margarine, glycaemic load, and fruits and vegetables [9].

To assess socioeconomic position, relative index of inequality (RII) based on education was used [27, 28]. RII measures the extent to which the occurrence of an outcome (e.g. death) varies by socio-economic position. RII considers the size of each study centre and the distributions of each level of education (i.e. none, primary, secondary, professional/technical, university or higher) within each study centre, thus minimising group differences across study centre.

### Outcome assessment

Information on vital status during follow-up, comprising of date and cause of death, was obtained through national or regional mortality registries in their respective countries or through active follow-up [29]. The 10th revision of the International Statistical Classification of Diseases and Related Health Problems (ICD-10) was used to define cancer mortality (i.e. ICD C00-C97 as the underlying cause of death) [30].

### Statistical analysis

#### Missing data

Of the 308,497 participants included in the study, 118,666 had missing values for at least one of the four HLI factors in either questionnaire. For these participants, we performed a multiple imputation by chained equation (MICE) by sex to iteratively impute the missing values, accounting for all HLI components and an a priori defined set of relevant covariates, i.e. age at follow-up questionnaire, study centre, height, RII, time between questionnaires (log-transformed), time to event or censorship (log-transformed), baseline diet score, and the all-cause mortality outcome. Menopausal status and use of hormone replacement therapy were also included for women. Missing data for covariates (i.e. 3.6% participants missing RII, 2.1% women missing hormone use) were also imputed. A total of 15 imputed datasets were generated with 20 iterations per imputation.

#### Hazard ratios

Associations between HLI changes and mortality outcomes were modelled with Cox proportional hazards regression using age as the underlying timescale, stratified by sex, centre, and age at recruitment in one-year increments to estimate hazard ratios (HR) and 95% confidence intervals (CI). Entry time was defined as age at follow-up questionnaire, and exit time was the age of death or loss/end of follow-up, whichever occurred first. Models were adjusted for HLI at baseline, baseline diet score, height, RII, and log time difference between questionnaires. In models for women, menopausal status and use of hormone replacement therapy were also included. The assumption of proportional hazards of HLI change was tested by Schoenfeld residuals, using the complete case data (*p* = 0.58 for improving HLI *p* = 0.74 for worsening HLI). Nonlinearity was assessed using restricted cubic splines for continuous HLI change. The log-likelihood of models with a linear term for the HLI change with a model with the linear term and the terms of cubic spline of HLI change to *χ*^2^ distribution with three degrees of freedom.

Subgroup analyses were performed by sex, baseline HLI categories (high [HLI = 11–16], medium [HLI = 9–10], low [HLI = 0–8]), SEP (estimated by a dichotomous variable representing the upper and lower 50th percentiles of RII, for low and high SEP, respectively), and smoking status (never vs current, removing the smoking component from HLI), as well as by country. Interaction with sex, baseline HLI categories, SEP, smoking status, and age of change (estimated as the average age between baseline and follow-up assessments) was tested with likelihood ratio test comparing the log-likelihood of models with and without the interaction terms to a chi-square distribution with degrees of freedom equal to the number of interaction terms. Analyses were performed on both the complete case dataset (results not shown) and the multiple imputation dataset. Parameter estimates from each imputed dataset were averaged out using the Rubin’s rule [31, 32] to account for uncertainty in the MICE.

As sensitivity analyses, the same models were fitted leaving out one HLI factor at a time to assess the contributions of each factor to the overall association. In the leave-one-out sub-analysis, the composite HLI scores were recalculated as the sum of the three remaining HLI components (e.g. when leaving smoking out, the HLI score was recalculated as the sum of alcohol, BMI, and physical activity scores, for a total maximum score of 12), and the models were adjusted for that factor at follow-up. Change scores were then re-calculated in the same way as in the main analysis. To address potential reverse causation, we also assessed associations after excluding the first 2 and 5 years of follow-up.

#### Rate advancement period

Rate advancement period (RAP) estimates were used to measure the impact of HLI change on all-cause mortality by quantifying the time by which mortality rates are advanced (or delayed) for participants with worsened (or improved) HLIs, compared to stable HLI [33, 34]. The methods of calculating RAP are described in detail elsewhere [34]. In brief, RAP estimates can be interpreted as the amount of time in years by which the rate of death is advanced amongst exposed individuals relative to unexposed individuals (e.g. RAP = 1 means the exposed individual reaches the same level of risk as an unexposed individual 1 year sooner). Follow-up time was used as the underlying time variable, with age at follow-up included as a covariate. RAP and respective 95% CI were estimated by dividing the log(HR) estimate for HLI change by the coefficient for age at follow-up.

Statistical tests were two-sided, and *p*-values < 0.05 were considered statistically significant. Analyses were performed with the ‘survival’ package in R V4.1.3 [35] and the ‘mi’ package in Stata/SE 17.0 [36].

## Results

### Participant characteristics

A total of 308,497 study participants (219,347 women and 89,150 men) were followed for a median (IQR) of 17.4 (15.3–19.2) years (5,221,718 total person-years), starting from recruitment until death or loss to follow-up. 21,696 deaths (8407 cancer-related) were documented (Table 1). Median (IQR) age at recruitment was 51.5 (45.5–57.6) years, and participants completed a follow-up questionnaire an average of 7.1 years after recruitment. Median (IQR) follow-up time between the second questionnaire and when participants exited the study was 9.6 (7.4–12.4) years. Overall, mean (SD) HLI change was 0.08 (2.06), with 41% improving (i.e. increasing HLI by more than one unit), 48% stable (i.e. HLI change within one unit), and 11% worsening (i.e. decreasing HLI by more than one unit). HLI changes were similar for men and women, but heterogeneous across countries (Fig. S2), with participants from Norway displaying the greatest negative mean (SD) change of − 0.98 (2.00) and participants from France with the greatest positive mean change of 1.17 (2.36). This might partially be explained by the mean baseline HLI, which was higher in Norway (11.1) than France (9.6). Changes in HLI scores (i.e. high, 12–16; medium, 9–11; low, 0–8) as well as for individual HLI component changes (score from 0 to 4) are shown in Sankey diagrams in Fig. S3. Country- and sex-specific HLI changes are shown in Fig. S4. Most changes in the positive direction were from smoking cessation, while most changes in the negative direction came from increased BMI. Baseline HLI (by tertiles) was evenly distributed across SEP, and HLI changes did not differ substantially for high and low SEP.

**Table 1:** Characteristics of the study population at recruitment by HLI change categories

	**HLI change category**			
	**Improve**	**Stable**	**Worsen**	**Overall**
***N***	**125,205**	**148,459**	**34,833**	**308,497**
**Person-years**	2,168,143	2,489,234	564,341	5,221,718
**All-cause deaths**	8790	10,351	2555	21,696
**Cancer deaths**	3553	3961	893	8407
**Age at recruitment [years]**	51.7 (8.6)	51.2 (9.1)	50.5 (9.6)	51.3 (8.96)
**Sex**				
Female	88,807 (70.9%)	105,924 (71.3%)	24,616 (70.7%)	219,347 (71.1%)
Male	36,398 (29.1%)	42,535 (28.7%)	10,217 (29.3%)	89,150 (28.9%)
**Education**				
None or primary	37,561 (30.0%)	43,629 (29.4%)	9254 (26.6%)	90,444 (29.3%)
Technical/professional or secondary	53,652 (42.9%)	62,792 (42.3%)	15,879 (45.6%)	132,323 (42.9%)
University or higher	32,012 (25.6%)	38,140 (25.7%)	8457 (24.3%)	78,609 (25.5%)
Not specified	1980 (1.6%)	3898 (2.6%)	1243 (3.6%)	7121 (2.3%)
**BMI [kg/m**^**2**^**]**	25.3 (4.32)	25.4 (4.19)	25.3 (3.66)	25.3 (4.19)
**Smoking status**				
Current	43,702 (34.9%)	34,808 (23.4%)	7175 (20.6%)	85,685 (27.8%)
Former	31,363 (25.0%)	41,668 (28.1%)	11,543 (33.1%)	84,574 (27.4%)
Never	50,140 (40.0%)	71,983 (48.5%)	16,115 (46.3%)	138,238 (44.8%)
**Alcohol intake [g/day]**	12.4 (16.9)	12.4 (17.3)	12.8 (17.7)	12.4 (17.2)
**METS recreational and household activity weekly**	78.7 (48.8)	83.2 (49.2)	85.2 (48.6)	81.7 (49.0)
**HLI at baseline**				
Low (0–8)	51,031 (40.8%)	28,129 (18.9%)	2848 (8.2%)	82,008 (26.6%)
Medium (9–11)	51,264 (40.9%)	56,448 (38.0%)	10,896 (31.3%)	118,608 (38.4%)
High (12–16)	22,910 (18.3%)	63,882 (43.0%)	21,089 (60.5%)	107,881 (35.0%)

Continuous variables expressed as mean (standard deviation). Categorical variables expressed as *n* (%)

Categories of HLI change defined as: ‘Improve’ (> 1 unit difference), ‘Stable’ (within ± 1 unit), and ‘Worsen’ (< 1 unit) of HLI scores between follow-up and baseline

### HLI change and mortality risk

**Fig. 2:**
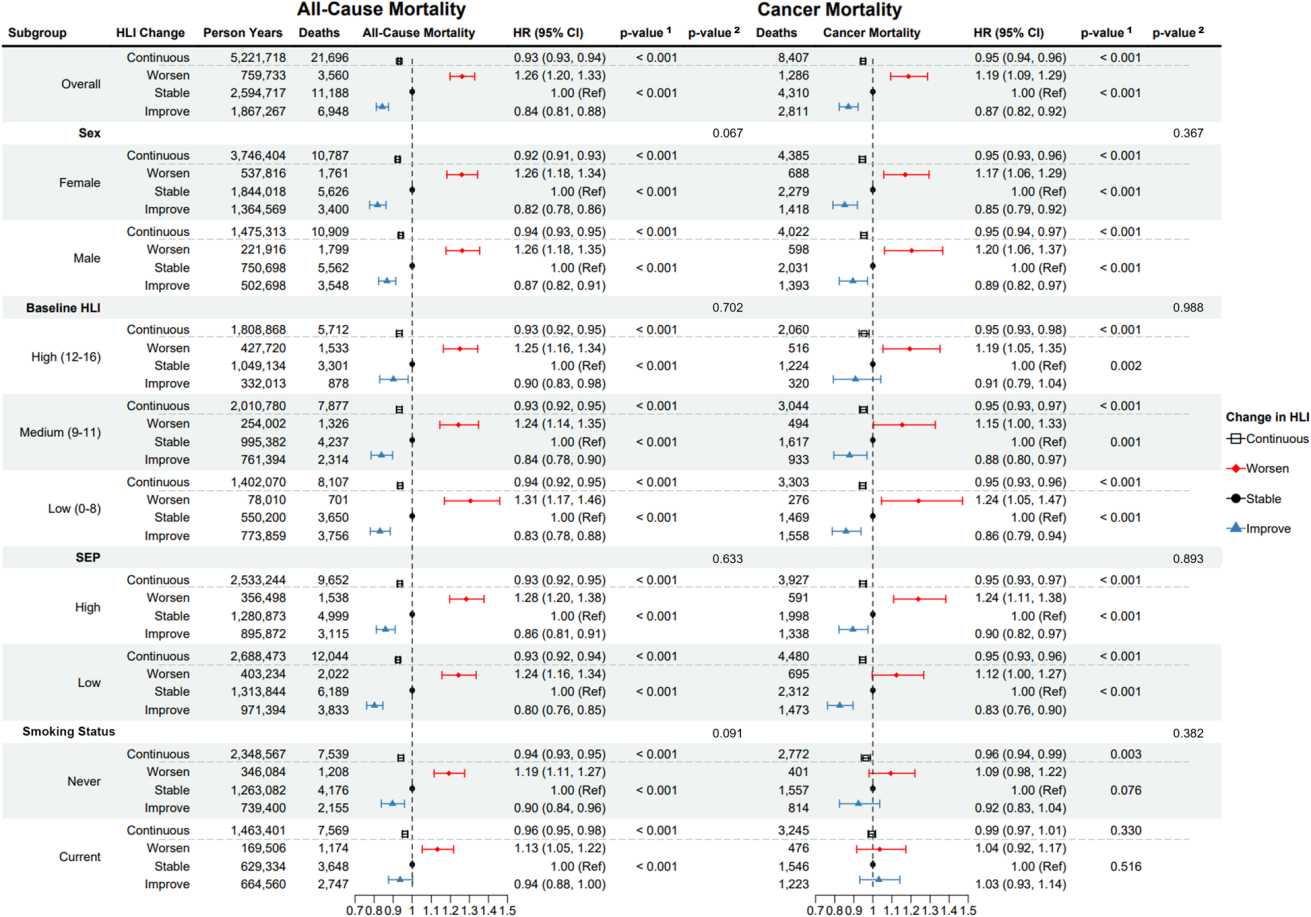
Forest plot of all-cause and cancer mortality hazard ratios (and 95% confidence intervals and *p*-values) for HLI change as continuous (per one-unit HLI change) and categorical (improve, increasing HLI by more than one unit; stable, within a one-unit change; worsen, decreasing HLI by more than one unit), overall and for subgroups, by sex, baseline HLI, socioeconomic position (SEP), and baseline smoking status

Results of the Cox regression model of HLI change on mortality, including subgroup analyses by sex, baseline HLI, SEP, and smoking status, are displayed in Fig. 2. A one-unit improvement in HLI was inversely associated with both all-cause and cancer mortality, with HR (95% CI) of 0.93 (0.93, 0.94) and 0.95 (0.94, 0.96), respectively. The associations observed were similar for men and women, and across subgroups. In the categorical analysis, improving HLI by more than one unit was associated with lower all-cause (HR: 0.84; 95% CI: 0.81, 0.88) and cancer (0.87; 0.82, 0.92) mortality compared to those whose HLIs remained stable, and worsening HLI by more than one unit was associated with an increase in all-cause (1.26; 1.20, 1.33) and cancer (1.19; 1.09, 1.29) mortality. Stronger associations were observed for lower baseline HLI for the categorical analysis (all-cause mortality HR: 1.31; 95% CI 1.17, 1.46 and HR: 0.83; 95% CI: 0.78, 0.88 for worsening and improving HLI respectively, compared to stable, for low baseline HLI; all-cause mortality HR: 1.25; 95% CI: 1.16, 1.34 and HR: 0.90; 95% CI: 0.83, 0.98 for worsening and improving HLI respectively, compared to stable, for high baseline HLI), although these differences were not significant. The low SEP subgroup saw stronger associations than the high SEP subgroup when improving HLI by more than one unit, compared to stable (all-cause mortality HR: 0.80; 95% CI: 0.76, 0.85 for low SEP; all-cause mortality HR: 0.86; 95% CI: 0.81, 0.91 for high SEP), while the high SEP subgroup had stronger associations when worsening HLI by more than one unit (all-cause mortality HR: 1.28; 95% CI: 1.20, 1.38 for high SEP; HR: 1.24; 95% CI: 1.16, 1.34 for low SEP), although these differences were not significant. No significant interactions were observed between HLI change and sex, baseline HLI, SEP, smoking status, or age of change. There were no substantial differences in associations across countries (Table S1) nor when considering other chronic conditions, such as cardiovascular disease or type-2 diabetes (results not shown). The same analyses were performed on complete cases and yielded similar results (results not shown).

**Fig. 3:**
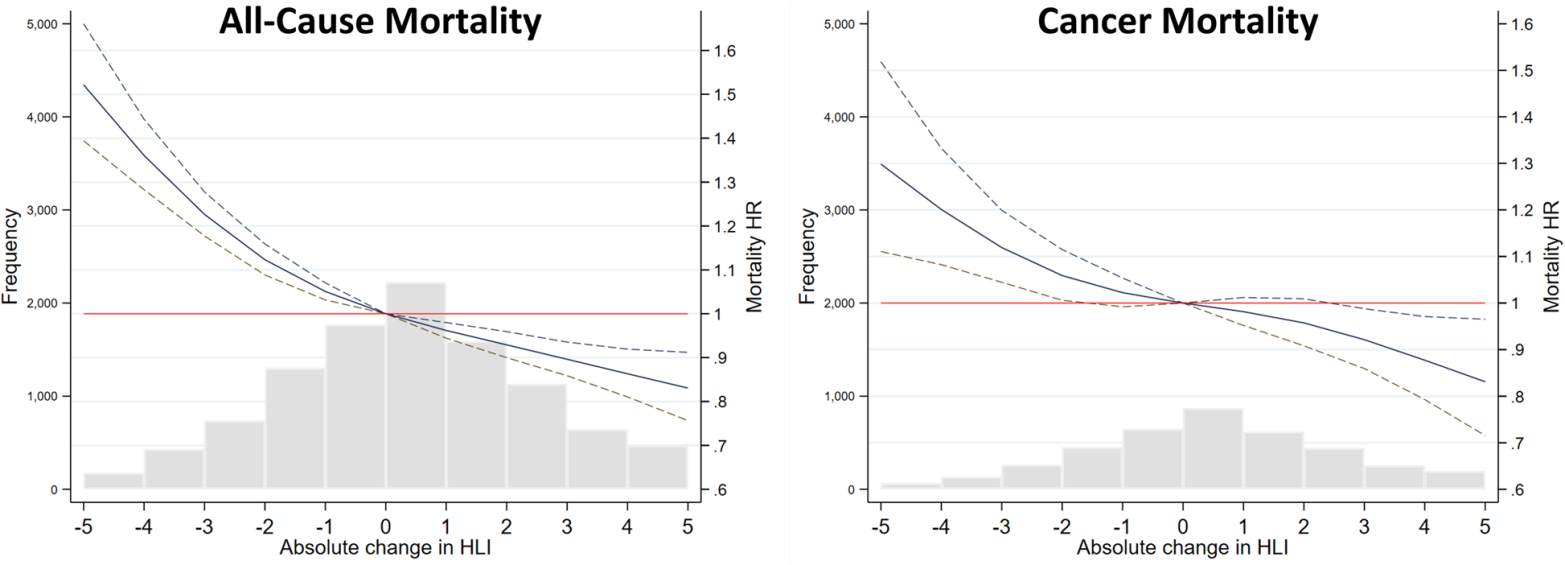
Cubic spline of all-cause and cancer mortality hazard ratios (HR^1^ 95% CI) by HLI change between follow-up and baseline assessments (complete case data). Histograms show the number of deaths

Additionally, with complete cases, continuous HLI change was related to mortality HRs using natural cubic splines to assess linearity (Fig. 3). Despite the associations appearing linear by visual inspection, the models indicated departure from linearity for both all-cause and cancer mortality (*p* < 0.001).

To address the potential reverse causality of changes in HLI due to unknown underlying health conditions, we also assessed the associations excluding observations in the first 2 and 5 years after follow-up. Associations remained virtually unchanged with these exclusions (Fig. S5).

**Fig. 4:**
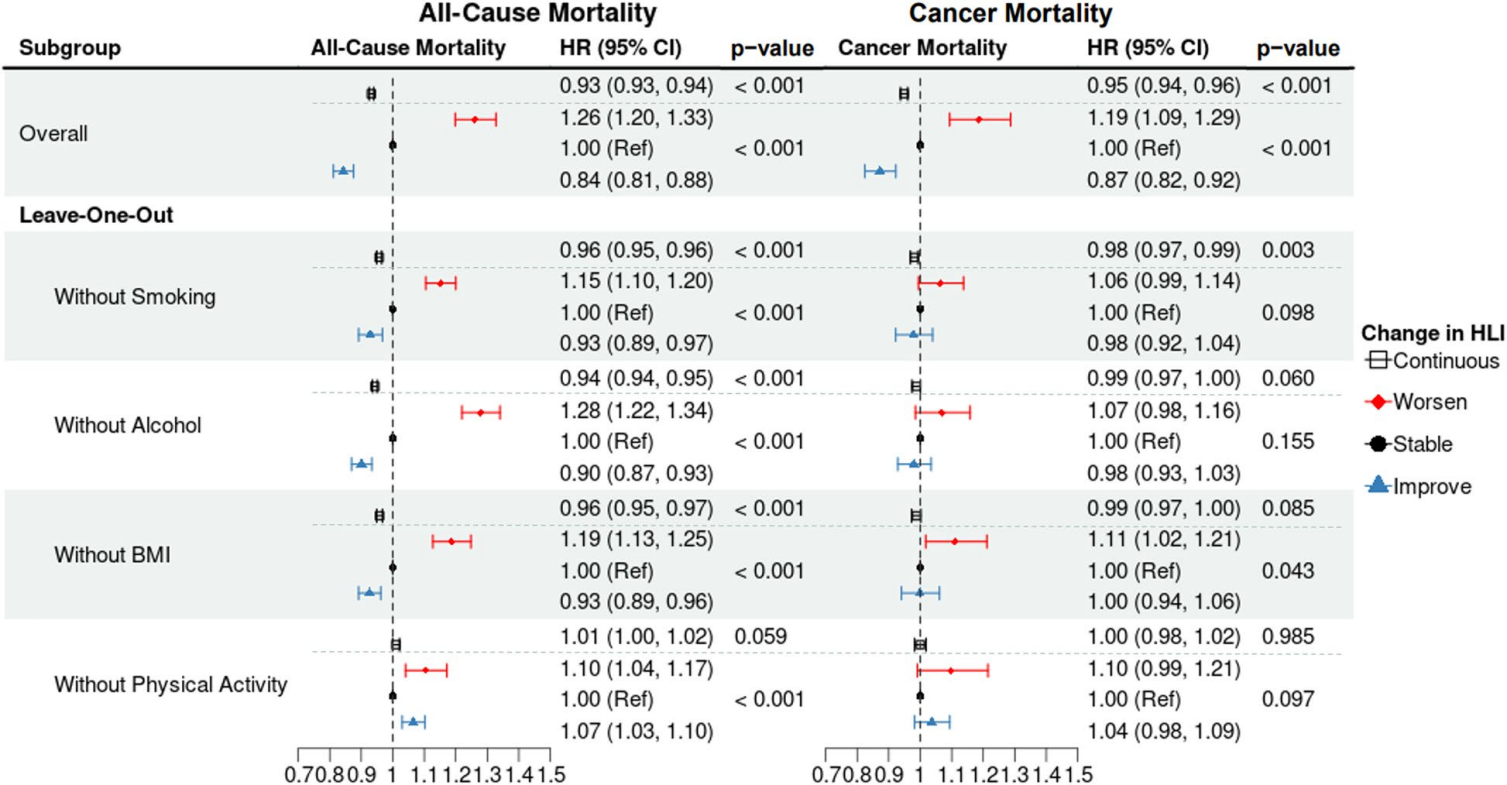
Forest plot of all-cause and cancer mortality hazard ratios (and 95% confidence intervals and *p*-values) for HLI change, leaving out one component at a time

In the leave-one-out analyses, the associations between HLI change and mortality were modelled, omitting one of the four lifestyle components at a time (Fig. 4). Associations between HLI change and all-cause mortality remained largely unchanged for all subgroups, except when omitting physical activity, where a positive association was observed with improving HLI, compared to stable (HR: 1.07; 95% CI: 1.03, 1.10). Associations between HLI change and cancer mortality were generally weakened towards the null when leaving out any one of the four components.

Associations between each of the HLI components and mortality were also assessed (Table S2). Improving alcohol score (i.e. decreasing alcohol intake) was positively associated with all-cause mortality (HR: 1.20; 95% CI: 1.13, 1.29). Additionally, both worsening and improving BMI score was associated with increased all-cause mortality, compared to stable (worsening HR: 1.11; 95% CI: 1.06, 1.16; improving HR: 1.08; 95% CI: 1.03, 1.13). These positive associations weakened towards the null after excluding the first 5 years of follow-up (results not shown).

### Rate advancement period

The impact of changing HLI on advancing the mortality rate was estimated through RAP, by sex and SEP (Table 2). A one-unit improvement in HLI corresponded to a − 0.50 (− 0.77, − 0.35) year RAP estimate. Compared to participants whose HLIs remained stable (within one unit) between baseline and follow-up, participants who improved their HLI by more than one unit had a − 1.19 (− 2.32, − 0.65) year RAP; participants who worsened their HLI by more than one unit had a 1.62 (1.44, 1.96) year RAP.

**Table 2:** Estimates of rate advancement period (RAP) for all-cause mortality by HLI change overall and by sex and socioeconomic position (SEP) (multiple imputation data)

	**Person-years**	***N***	**Deaths**	**RAP (95% CI) [years]**		
				**Continuous**	**HLI change**	
					**Worsen vs. stable**	**Improve vs. stable**
**Overall**	5,221,718	308,497	21,696	− 0.50 (− 0.77, − 0.35)	1.62 (1.44, 1.96)	− 1.19 (− 2.33, − 0.65)
**Sex**						
Female	3,746,404	219,347	10,787	− 0.55 (− 1.00, − 0.35)	1.84 (1.52, 2.76)	− 1.40 (− 3.47, − 0.65)
Male	1,475,314	89,150	10,909	− 0.46 (− 0.95, − 0.27)	1.42 (1.25, 1.90)	− 1.06 (− 3.53, − 0.38)
**SEP**						
High	2,668,002	158,823	10,292	− 0.54 (− 1.21, − 0.30)	1.88 (1.50, 3.50)	− 1.38 (− 6.13, − 0.47)
Low	2,553,716	149,674	11,404	− 0.48 (− 0.83, − 0.31])	1.47 (1.30, 1.86)	− 1.10 (− 2.46, − 0.52)

Categories of HLI change defined as: ‘Improve’ (> 1 unit difference), ‘Stable’ (within ± 1 unit), and ‘Worsen’ (< 1 unit) of HLI scores between follow-up and baseline

## Discussion

Using data from longitudinal assessments in the EPIC cohort, this study focused on the relationship between lifestyle changes and mortality during adulthood. Improving lifestyle behaviours was inversely associated with all-cause and cancer mortality, while worsening lifestyle behaviours was associated with increased mortality. Lifestyle changes were measured as the difference between baseline and follow-up HLI, integrating information on smoking status, alcohol intake, BMI, and physical activity. Improving HLI by more than one unit was associated with a 16% reduction in all-cause mortality and a 13% reduction in cancer mortality, compared to no change, while worsening HLI by more than one unit was associated with a 26% increase in all-cause mortality and an 19% increase in cancer mortality.

In this study, we investigated whether associations between HLI change and mortality differed by sex, HLI score at baseline, SEP, and smoking status. Although improving HLI, compared to a stable HLI, was slightly more strongly associated with all-cause mortality in women (HR: 0.82; 95% CI: 0.78, 0.86) than men (0.87; 0.82, 0.91) and amongst participants with poor lifestyle profiles at baseline (0.83; 0.78, 0.88) than for those with healthy lifestyles at baseline (0.90; 0.83, 0.98), the observed relationships were of similar magnitude by sex, by baseline HLI, amongst participants with low and high SEP, and by smoking status. Interestingly, changing HLI was inversely associated with cancer mortality amongst never smokers, but not amongst current smokers, once more highlighting the need for smokers to quit smoking [4].

In sensitivity analyses removing one component of the HLI score at a time, we found that associations of HLI change with all-cause mortality were generally weakened, and, after leaving out any one of the four components, HLI changes were not related to cancer mortality. These results suggest the importance of examining lifestyle factors jointly to quantify their impact on all-cause and cancer mortality.

Estimates of RAP were used to quantify the time dimension by which mortality is impacted by lifestyle changes. For this analysis, RAPs were computed for all-cause and cancer mortality assuming a linear relationship with age [33]. Compared to stable HLI, worsening HLI by more than one unit anticipated the risk of death by 1.62 (95% CI: 1.44, 1.96) years and improving HLI by more than one unit delayed the risk of death by 1.19 (0.65, 2.32) years. Caution must be used, however, in the interpretation of RAPs as they are often misinterpreted as the difference in mean survival time or the time by which a survival curve is shifted between exposed and unexposed participants [34].

To the best of our knowledge, only three other studies have evaluated changes in lifestyle behaviours on mortality using longitudinal data from national-level cohorts [17–19]. In a cohort of over 50,000 Chinese participants, lifestyle trajectories, measuring smoking status, alcohol intake, physical activity, sedentary behaviours, and salt intake, were related to all-cause mortality [18]. Trajectories expressing improving and worsening lifestyles were inversely and positively associated with all-cause mortality, compared to a stable lifestyle, with HR (95% CI) of 0.80 (0.70, 0.96) and 1.44 (1.13, 1.83), respectively. These associations were in line with the estimates of our study. In the Nurse’s Health Study (NHS) and the Health Professionals Follow-up Study (HPFS), trajectories of individual lifestyle factors (i.e. BMI, smoking status, diet, physical activity, alcohol intake) were evaluated separately in over 85,000 participants in relation to all-cause mortality and longevity, defined as surviving to age 85 or older [17]. Maximum longevity and the lowest risk of death were achieved by participants who maintained a low-stable BMI, who had a medium-stable alcohol intake, had a medium-increase physical activity, who never smoked or who were light-smokers and quit smoking, and who had a high-increase pattern for diet quality. Compared to the healthiest profile, combining the trajectories of all individual lifestyle factors, participants with the least healthy profile were 70% (95% CI: 68%, 71%) less likely to achieve longevity. Lastly, a study followed over 5000 Dutch adults over 5 years, collecting data on diet, physical activity, smoking status, and alcohol intake [19]. Each lifestyle behaviour was dichotomised (i.e. healthy, unhealthy) and summed to make a healthy lifestyle score; participants either improved, worsened, or remained stable. This study reported similar estimates to our own; however, improving lifestyle did not reach statistical significance whereas worsening did. We similarly found strong associations with HLI change and mortality in those whose HLI score worsened.

In our study, analyses of the four HLI components separately showed that reducing alcohol intake was associated with a 5% increase in the all-cause mortality HR per unit increase in the alcohol score from baseline to follow-up, while no associations were observed for cancer mortality. This result may be explained by the inverse association observed between alcohol intake and fatal coronary heart diseases [37]. In addition, an unknown proportion of participants who reduced their alcohol intake might have developed morbid conditions, ultimately related to the occurrence of fatal events, by means of reverse causation. Indeed, when excluding the first 5 years of follow-up, the all-cause mortality HR of increasing alcohol consumption was not statistically significant (HR: 1.02; 95% CI: 0.99, 1.04). Additionally, improving and worsening BMI scores were positively associated with all-cause mortality, with HR (95% CI) of 1.08 (1.03, 1.13) and 1.11 (1.06, 1.16), respectively, compared to maintaining stable BMI between baseline and follow-up. These findings support a U-shaped relationship between BMI changes and all-cause mortality, as observed previously [17, 38, 39]. A study on healthy lifestyle changes in a Chinese cohort observed an increased rate of all-cause mortality in participants whose BMIs either increased or decreased, compared to those whose BMIs remained stable [18].

This study has several strengths. It is the largest observational study using longitudinal data to relate combined multifactorial lifestyle changes to mortality. Drawing from a large-scale population-based prospective study with over 5 million person-years from over 300,000 participants across multiple centres in 9 countries, this study exploited a large sample size to perform multiple stratified analyses with sufficient statistical power. Additionally, the use of a composite HLI score, rather than only investigating each individual component separately, allowed for a more representative analysis of lifestyle changes on mortality, as the ensemble of (healthier or unhealthier) lifestyle behaviours are often related to one another.

Our study also has limitations. Combining multiple lifestyle factors into a single score might mask relevant behavioural changes. For example, smoking cessation might be associated with weight gain, which may result as a net zero change in HLI score. Also, in the computation of the HLI, each lifestyle component was given equal weight despite evidence supporting a stronger association between smoking and mortality, than between BMI and mortality [40, 41].

As in all large-scale prospective studies, the collection and harmonisation of heterogenous data across multiple study centres is a challenge. At this time, we were unable to include a dietary component in the HLI, despite its observed role in cancer mortality [12], as the harmonisation of dietary data at follow-up in EPIC is currently underway. The HLI used in this study also does not include exposure information on potentially relevant lifestyle factors, like sleep or stress, which may also play an important role in mortality [42, 43]. Additionally, to estimate SEP, we calculated the RII based on education level, as this was the only common variable across the 23 study centres. Although RII has been shown to be strongly associated with mortality and cancer, even after accounting for lifestyle factors [44, 45], it remains a limited measure. A recent evaluation of the US NHANES and UK Biobank studied the associations of healthy lifestyle and SEP with mortality, which combined educational level, family income, occupation, and health insurance to measure SEP, and found stronger associations between healthy lifestyle and mortality were observed amongst low SEP participants of compared to high SEP participants in UK Biobank but not in US NHANES [22]. Additional metrics of SEP may bolster the initial findings of our present study.

Lastly, it is possible that some lifestyle changes measured in these assessments were either prone to measurement error or were not made as a voluntary decision by study participants but rather in response to underlying health conditions. To address the role of unknown underlying health conditions, the first few years after exposure assessments were excluded in a sensitivity analysis. Overall associations between HLI change and mortality remained virtually unchanged after these exclusions.

## Conclusions

Using longitudinal data from a large European cohort, this study brings novel evidence on the benefits of adopting healthier lifestyle choices on all-cause and cancer mortality. Making healthier lifestyle changes in adulthood is associated with lower all-cause and cancer mortality as well as a delayed risk of death. Conversely, worsening lifestyle behaviours are associated with increased rates of mortality and an anticipated risk of death.

### Supplementary Information

**Additional file 1:** **Fig. S1.** Calculation of the Healthy Lifestyle Index (HLI) score. **Fig. S2.** Country- and sex-specific mean HLI change between follow-up and baseline. **Fig. S3.** Sankey diagram for HLI categories showing HLI changes between baseline and follow-up assessments, and for each HLI component. **Fig. S4.** Country-specific HLI mean score difference between follow-up and baseline overall and for each HLI component. **Fig. S5.** Forest plot of all-cause and cancer mortality hazard ratios (and 95% confidence intervals) for HLI change, excluding observations up to the first 2 and 5 years after follow-up lifestyle questionnaire (washout). **Table S1.** All-cause and cancer mortality hazard ratios (and 95% confidence intervals) for HLI change by country. **Table S2.** All-cause and cancer mortality hazard ratios (and 95% confidence intervals) for HLI change by HLI component.

## Data Availability

Access to the EPIC data is governed by the EPIC access policy, as detailed in https://epic.iarc.fr/docs/EPIC-Europe_AccessPolicy_01Feb2023.pdf. Please contact the corresponding author, Dr Pietro Ferrari, for more information.

## Abbreviations

BMI: Body mass index
CI: Confidence interval
EPIC: European Prospective Investigation into Cancer and Nutrition
HLI: Healthy lifestyle index
HR: Hazard ratio
HPFS: Health Professionals Follow-up Study
IARC: International Agency for Research on Cancer
ICD: International Classification of Diseases
IQR: Interquartile range
MICE: Multiple imputation by chained equation
NHANES: National Health and Nutrition Examination Survey
NHS: Nurse’s Health Study
RAP: Rate advancement period
RII: Relative inequality index
SD: Standard deviation
SEP: Socio-economic position
