# Supplementary material for: Healthy lifestyle change and all-cause and cancer mortality in the European Prospective Investigation into Cancer and Nutrition cohort
Source: BMC Med. 2024 May 29;22:210. doi: 10.1186/s12916-024-03362-7 (PMC11134634; doi:10.1186/s12916-024-03362-7)
Supplement: Supplementary file 1 — Additional file 1: Fig. S1. Calculation of the Healthy Lifestyle Index (HLI) score. Fig. S2. Country- and sex-specific mean HLI change between follow-up and baseline. Fig. S3. Sankey diagram for HLI categories showing HLI changes between baseline and follow-up assessments, and for each HLI component. Fig. S4. Country-specific HLI mean score difference between follow-up and baseline overall and for each HLI component. Fig. S5. Forest plot of all-cause and cancer mortality hazard ratios (and 95% confidence intervals) for HLI change, excluding observations up to the first 2 and 5 years after follow-up lifestyle questionnaire (washout). Table S1. All-cause and cancer mortality hazard ratios (and 95% confidence intervals) for HLI change by country. Table S2. All-cause and cancer mortality hazard ratios (and 95% confidence intervals) for HLI change by HLI component. [file 12916_2024_3362_MOESM1_ESM.docx]

**Figure S1.** Calculation of the Healthy Lifestyle Index (HLI) score.


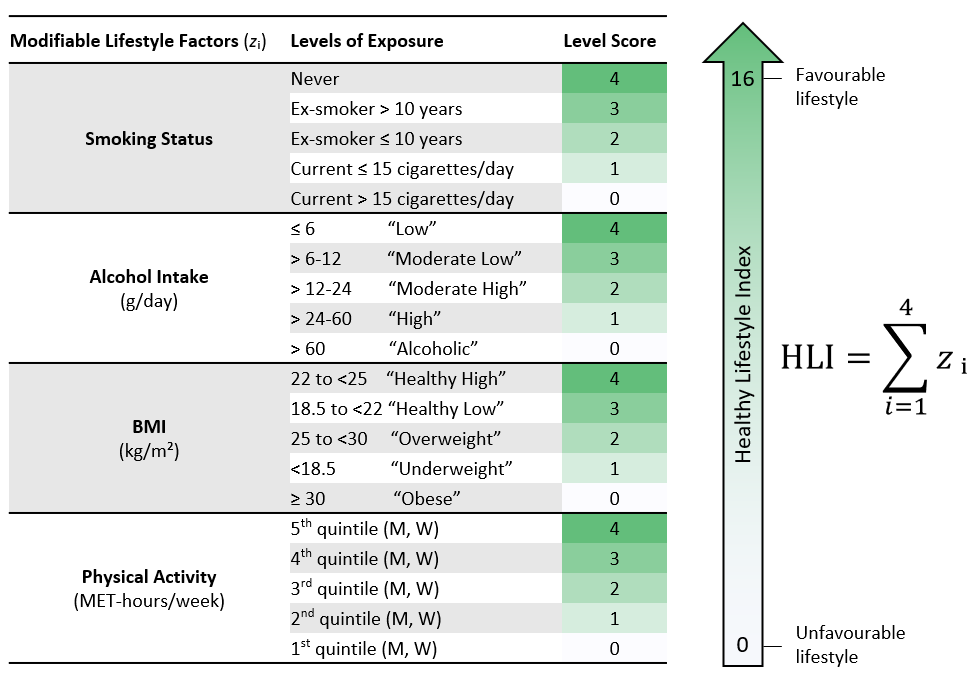


**Figure S2.** Country- and sex-specific mean HLI change between follow-up and baseline, mean age at baseline, mean HLI at baseline, mean HLI at follow-up and mean time difference between follow-up and baseline.


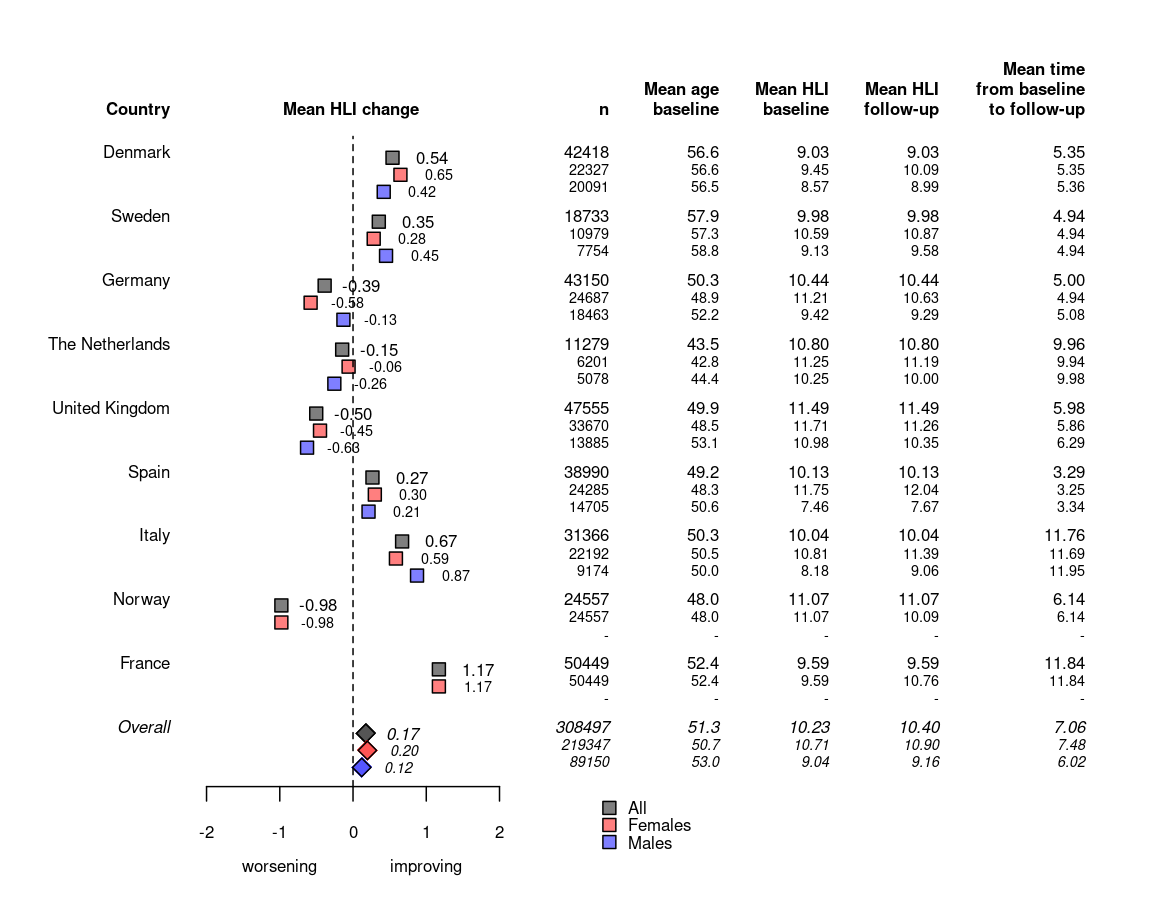


**Figure S3.** Sankey diagram for HLI categories (low, 0-8; medium, 9-11; high, 12-16 units) showing HLI changes between baseline and follow-up assessments, and for each HLI component.


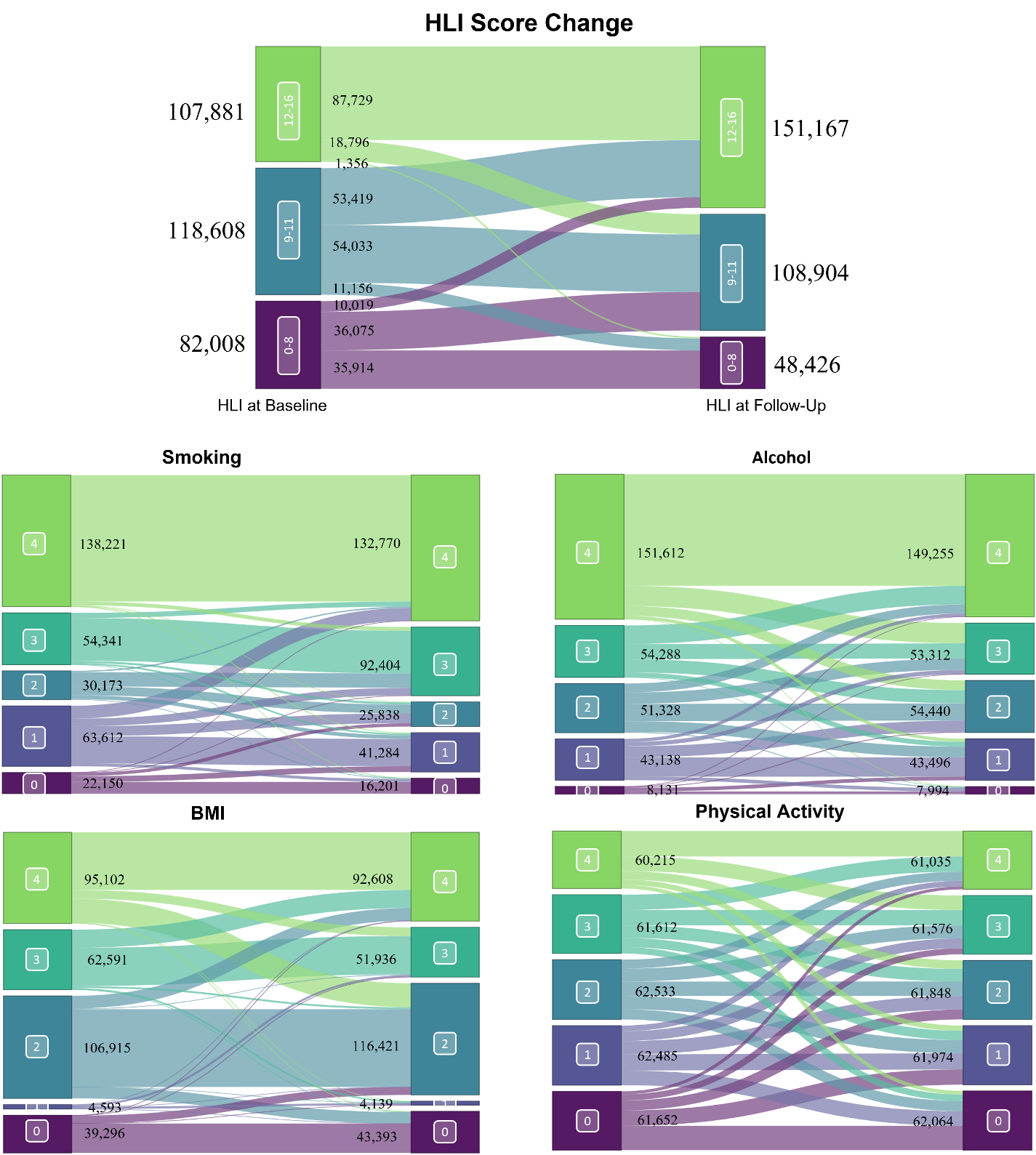


**Figure S4.** Country-specific HLI mean score difference between follow-up and baseline overall and for each HLI component. Positive changes indicate lifestyle improvements over time.


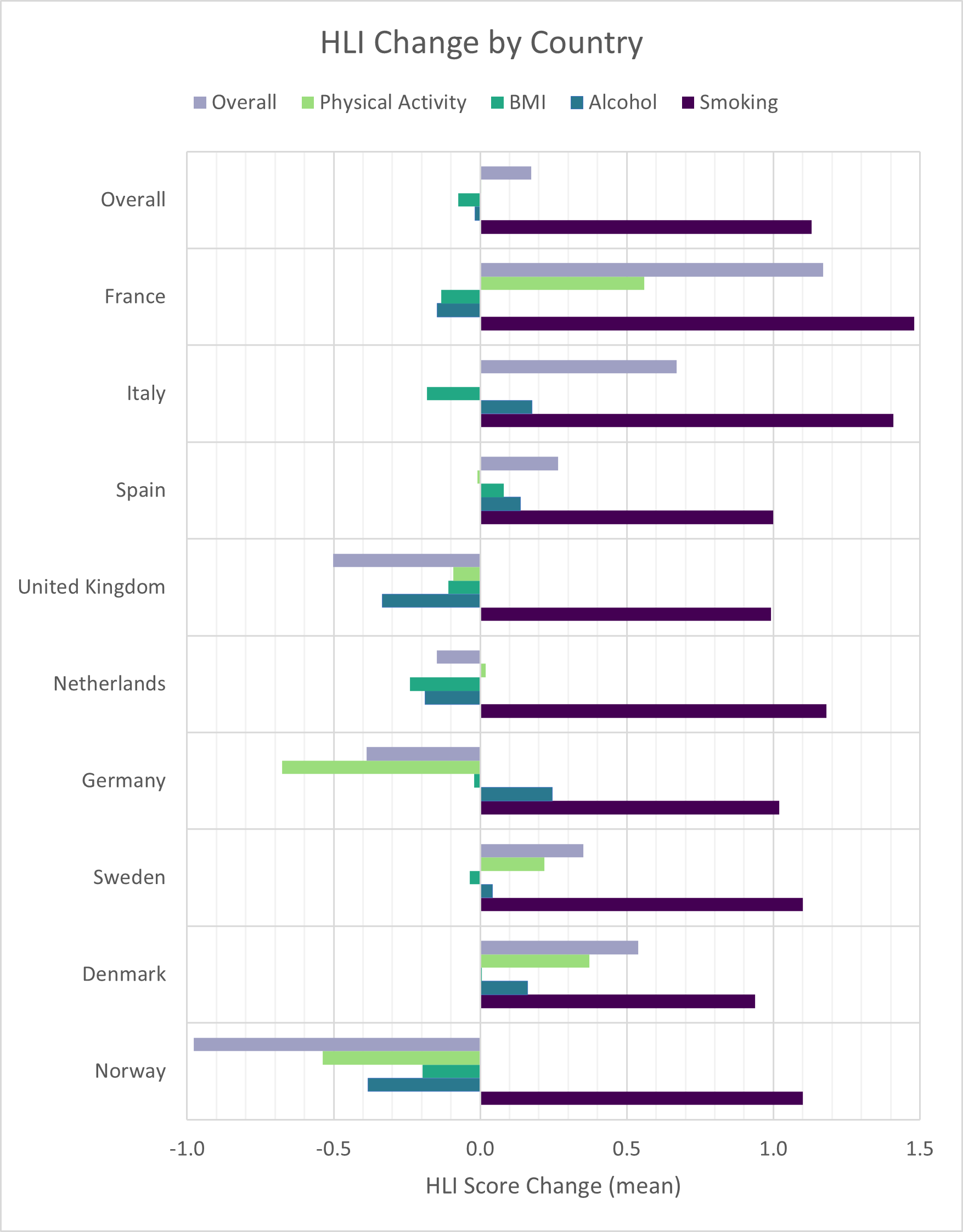


**Figure S5**. Forest plot of all-cause and cancer mortality hazard ratios (and 95% confidence intervals) for HLI change, excluding observations up to the first 2 and 5 years after follow-up lifestyle questionnaire (washout) (multiple imputation data).


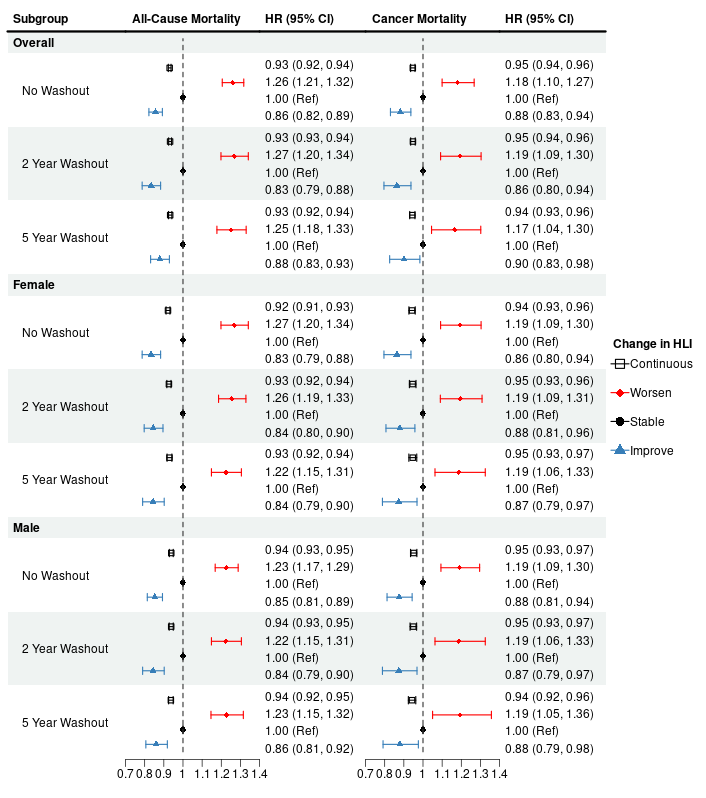


**Table S1**. All-cause and cancer mortality hazard ratios (and 95% confidence intervals) for HLI change by country (multiple imputation data).

|  |  |  | **All-Cause Mortality** | | | | | **Cancer Mortality** | | | | |
| --- | --- | --- | --- | --- | --- | --- | --- | --- | --- | --- | --- | --- |
|  | **Person-Years** | **N** | **All Deaths** | **HR (95% CI)** | | | | **Cancer Deaths** | **HR (95% CI)** | | | |
|  |  |  |  | **Continuous** | **Categorical** | | |  | **Continuous** | **Categorical** | | |
|  |  |  |  |  | **Worsen** | **Stable** | **Improve** |  |  | **Worsen** | **Stable** | **Improve** |
| France | 990,581 | 50,449 | 1,483 | **0.92 (0.90, 0.94)** | **1.23 (1.01, 1.51)** | 1 (ref) | **0.73 (0.64, 0.82)** | 455 | **0.91 (0.87, 0.96)** | 1.33 (0.91, 1.93) | 1 (ref) | **0.71 (0.57, 0.88)** |
| Italy | 495,704 | 31,366 | 505 | **0.93 (0.89, 0.98)** | 1.15 (0.81, 1.63) | 1 (ref) | 0.81 (0.65, 1.01) | 249 | 0.94 (0.88, 1.01) | 1.16 (0.72, 1.86) | 1 (ref) | 0.84 (0.62, 1.15) |
| Spain | 724,102 | 38,990 | 3,051 | **0.95 (0.93, 0.97)** | **1.34 (1.17, 1.53)** | 1 (ref) | **0.86 (0.80, 0.94)** | 1,387 | **0.95 (0.92, 0.97)** | **1.36 (1.12, 1.67)** | 1 (ref) | 0.90 (0.80, 1.01) |
| United Kingdom | 818,226 | 47,555 | 4,614 | **0.95 (0.94, 0.97)** | **1.26 (1.16, 1.36)** | 1 (ref) | 0.95 (0.88, 1.02) | 1,306 | 0.98 (0.95, 1.01) | 1.15 (0.99, 1.34) | 1 (ref) | 0.98 (0.85, 1.12) |
| Netherlands | 196,283 | 11,279 | 341 | 0.94 (0.89, 1.00) | **1.61 (1.15, 2.25)** | 1 (ref) | 1.14 (0.85, 1.50) | 153 | 0.95 (0.87, 1.03) | 1.63 (0.98, 2.72) | 1 (ref) | 1.12 (0.74, 1.69) |
| Germany | 596,544 | 43,150 | 1,981 | **0.92 (0.90, 0.94)** | **1.31 (1.15, 1.49)** | 1 (ref) | **0.84 (0.76, 0.94)** | 848 | **0.94 (0.91, 0.98)** | 1.14 (0.93, 1.40) | 1 (ref) | 0.85 (0.73, 1.00) |
| Sweden | 347,472 | 18,733 | 4,149 | **0.93 (0.91, 0.95)** | **1.27 (1.14, 1.42)** | 1 (ref) | **0.86 (0.78, 0.94)** | 1,474 | **0.96 (0.93, 0.98)** | **1.21 (1.02, 1.45)** | 1 (ref) | 0.91 (0.79, 1.04) |
| Denmark | 708,602 | 42,418 | 5,128 | **0.93 (0.91, 0.94)** | **1.19 (1.07, 1.32)** | 1 (ref) | **0.80 (0.74, 0.87)** | 2,277 | **0.94 (0.92, 0.97)** | 1.13 (0.96, 1.33) | 1 (ref) | **0.84 (0.75, 0.95)** |
| Norway | 344,205 | 24,557 | 444 | **0.90 (0.85, 0.95)** | **1.43 (1.09, 1.87)** | 1 (ref) | 0.91 (0.63, 1.31) | 258 | 0.97 (0.90, 1.05) | 1.07 (0.75, 1.53) | 1 (ref) | 0.93 (0.60, 1.45) |

**Table S2**. All-cause and cancer mortality hazard ratios (and 95% confidence intervals) for HLI change by HLI component (0-4) (multiple imputation data).

|  | **All-Cause Mortality** | | | | **Cancer Mortality** | | | |
| --- | --- | --- | --- | --- | --- | --- | --- | --- |
|  | **HR (95% CI)** | | | | **HR (95% CI)** | | | |
|  | **Continuous** | **Categorical** | | | **Continuous** | **Categorical** | | |
|  |  | **Worsen** | **Stable** | **Improve** |  | **Worsen** | **Stable** | **Improve** |
| Smoking | **0.93 (0.90, 0.95)** | **1.22 (1.01, 1.46)** | 1 (ref) | **0.82 (0.78, 0.86)** | **0.94 (0.90, 0.97)** | 1.04 (0.76, 1.44) | 1 (ref) | **0.80 (0.74, 0.86)** |
| Alcohol | **1.05 (1.04, 1.07)** | 0.99 (0.93, 1.05) | 1 (ref) | **1.20 (1.13, 1.29)** | 1.01 (0.98, 1.04) | 1.09 (0.99, 1.21) | 1 (ref) | 1.11 (1.00, 1.23) |
| BMI | **0.98 (0.97, 0.99)** | **1.11 (1.06, 1.16)** | 1 (ref) | **1.08 (1.03, 1.13)** | 0.98 (0.95, 1.00) | 1.10 (1.00, 1.18) | 1 (ref) | 1.01 (0.94, 1.09) |
| Physical Activity | **0.91 (0.89, 0.92)** | **1.30 (1.24, 1.35)** | 1 (ref) | **0.86 (0.82, 0.90)** | **0.98 (0.96, 0.99)** | 1.08 (1.00, 1.17) | 1 (ref) | 0.97 (0.90, 1.04) |
